# Impaired Coronary Endothelial Vasorelaxation in a Preclinical Model of Peripheral Arterial Insufficiency

**DOI:** 10.15436/2378-6914/15/010

**Published:** 2015-06-29

**Authors:** A.A Arce-Esquivel, A.K Bunker, G.H Simmons, H.T Yang, M.H Laughlin, R.L Terjung

**Affiliations:** 1 Department of Biomedical Sciences, College of Veterinary Medicine, University of Missouri, Columbia, Missouri; 2 Department of Medical Pharmacology and Physiology, College of Medicine, University of Missouri, Columbia, Missouri; 3 Dalton Cardiovascular Research Center, University of Missouri, Columbia, Missouri; 4 Department of Health and Kinesiology, The University of Texas at Tyler, Tyler, Texas; 5 Morningside College, Sioux City, Iowa

**Keywords:** Coronary dysfunction, Peripheral artery disease, Exercise, Miniature swine

## Abstract

The present study was designed to determine whether adult swine with peripheral artery insufficiency (PAI) would exhibit vascular dysfunction in vessels distinct from the affected distal limbs, the coronary conduit arteries. Moreover, we sought to evaluate the effect of exercise training on coronary vasomotor function in PAI. Eighteen female healthy young Yucatan miniature swine were randomly assigned to either occluded exercise trained (Occl-Ex, n=7), or occluded-sedentary (Occl-Sed, n=5), or non-occluded, non-exercised control (Non-Occl-Con, n=6) groups. Occl-Ex pigs were progressively trained by running on a treadmill (5days/week, 12 weeks). The left descending artery (LAD) and left circumflex (LCX) coronary arteries were harvested. Vasorelaxation to adenosine diphosphate (ADP), bradykinin (BK), and sodium nitro-prusside (SNP) were assessed in LAD’s; while constrictor responses to phenylephrine (PE), angiotensin II (Ang II), and endothelin-1 (ET-1) were assessed in LCX’s. Vasorelaxation to ADP was reduced in LADs from Occl-Sed and Occl-Ex pigs (*P*<0.001) as compared to Non-Occl-Con pigs; however, Occl-Ex pigs exhibited partial recovery (*P*<0.001) intermediate to the other two groups. BK induced relaxation was reduced in LADs from Occl-Ex and Occl-Sed pigs (*P*<0.001), compared to Non-Occl-Con, and exercise modestly increased responses to BK (*P*<0.05). In addition, SNP, PE, Ang II, and ET-1 responses were not significantly different among the groups. Our results indicate that ‘simple’ occlusion of the femoral arteries induces vascular dysfunction in conduit vessels distinct from the affected hindlimbs, as evident in blunted coronary vasorelaxation responses to ADP and BK. These findings imply that PAI, even in the absence of frank atherogenic vascular disease, contributes to vascular dysfunction in the coronary arteries that could exacerbate disease outcome in patients with peripheral artery disease. Further, regular daily physical activity partially recovered the deficit observed in the coronary arteries.

## Introduction

Peripheral artery disease (PAD) impedes blood flow typically to the lower extremities leading to a mismatch of oxygen delivery that becomes more evident during physical activity^[1,2]^. PAD is a major cause of disability, loss of work, and lifestyle changes in the United States^[1]^. It affects approximately 8 to 12 million persons in the country^[1]^, and its prevalence increases with advancing age, as approximately 12 to 20% of people older than 70 years suffer from this condition^[3]^.

One half of all PAD patients older than 55 years are asymptomatic; whilst among the symptomatic, 40% experience intermittent claudication, the most common clinical manifestation of PAD, and 10% have limb ischemia at rest^[2]^. The impaired perfusion observed in PAD can foster functional and structural modifications in the affected limb (e.g., vascular and skeletal muscle adaptations) which attempt to ameliorate the symptoms^[4]^. This is especially true if the patients become physically active^[4]^. However, PAD patients also exhibit decreased nitric oxide (NO) activity that is associated with an increased production of vasoconstrictors and sympathetic outflow^[5,6]^.

PAD patients are reported to have increased incidence of coronary artery disease (CAD)^[7]^ with increased mortality due to cardiovascular disease, especially CAD (20 to 40% increased risk of a myocardial infarction), as well as a 6.6-fold higher overall mortality than non-PAD patients^[8]^. Interestingly, in patients with concomitant CAD and PAD the presence of peripheral inflammation has been postulated to contribute to coronary endothelial dysfunction, mostly due to a reduction in NO bioavailability^[9]^. Indeed, many conceive that the association of PAD with CAD is simply the result of generalized atherosclerosis progressing in both arterial trees. However, an estimated 35 to 50% of all patients with PAD do not exhibit CAD or cerebral vascular disease (CVD)^[10,11]^. There are studies reporting a widespread incidence of normal coronary arteries among PAD patients^[12–14]^. For instance, coronary angiography was found to be normal in 85% of symptomatic PAD patients (n=53; mean age 54y)^[12]^. Duran *et al*.^[13]^ reported that coronary artery angiography was within normal limits in 28% of patients with PAD (n=213; mean age 61y). Furthermore, the study by Hertzer *et al.*^[14]^ found that CAD was present in only 270 of 468 elderly patients (58%) with PAD who were living in a long-term health care facility. Nonetheless, these PAD patients in the absence of frank CAD and CVD have the same markedly increased morbidity and mortality as those PAD patients with attendant central vascular disease^[15]^. It is possible that the ischemic limb muscles in PAD release substances and/or initiate neural reflex responses systemically that lead to coronary artery dysfunction and potentially greater susceptibility to CAD. It is well documented that exercise training improves walking ability and reduces cardiovascular mortality and morbidity of claudicant patients^[16,17]^. Indeed, Sakamoto *et al.*^[17]^ reported that supervised exercise training reduced overall cardiovascular mortality by 52% and morbidity by 30% among PAD patients. Based on this background, the present study was designed to determine whether in an atherosclerosis free, preclinical animal model of peripheral artery insufficiency (PAI) bilateral occlusion of the femoral arteries would lead to vascular dysfunction within a vascular bed, the coronary conduit vessels, distant from the affected hindlimb vasculature. This animal model of PAI exhibits limb blood flow that is non-ischemic at rest, but is impaired during exercise^[18,19]^, similar to flow patterns seen with intermittent claudication. In addition, we sought to evaluate the effect of exercise training on coronary endothelial function in this animal model of PAI. Exercise training has been shown to increase endothelial-dependent vasorelaxation of peripheral vessels in patients with intermittent claudication^[20]^, and in patients with CAD^[21]^. Thus, we hypothesized that a preclinical animal model of PAI would exhibit coronary dysfunction and that this would be ameliorated by exercise.

## Methods

### Experimental animals

Healthy young female Yucatan miniature swine (n=18; 8–10months old, and weighing 30–40 kg), a breed relatively resistant to atherogenesis^[22–25]^, were used in this study. The pigs were housed in the animal care facility in rooms maintained at 20–23°C with a 12h: 12h light-dark cycle. They were fed a normal diet (Purina Laboratory 5082 Mini-Pig Breeder Chow) with *ad libitum* access to water. All experimental protocols were approved by the Institutional Animal Care and Use Committee of the University of Missouri.

### Surgical procedure

Animals were pre-anesthetized with ketamine (35 mg/ kg)-xylazine (2.25 mg/kg) intramuscular injection. A tracheal tube was inserted, and secured. Pigs were anesthetized by using 2% isoflurane gas with a closed circuit anesthesia machine and an auto-respirator (Ohmed 7000). Surgical areas were cleaned with warm soap water, sterilized with Betadine and alcohol. A 4cm incision was made on the pig’s neck to expose left carotid artery. A medical perfusion catheter (Original Perfusor-Leitung, B Braun Melsungen AG, Aesculap, Inc. Center Valley, PA) was inserted via the carotid artery and advanced into the thoracic aorta for blood pressure monitoring. Blood pressure, respiration rate, heart rate, ECG, and body temperature were monitored during the surgery procedure. A 4 cm longitudinal skin incision was made in each groin area above the femoral artery which was occluded with 2–0 surgical silk at 2 cm distal from the inguinal ligament. The femoral occlusion animal model employed in this experiment has been used extensively by our laboratory^[19]^.

### Training protocol

At the beginning of the study all pigs were familiarized with walking on a motorized treadmill for 5 days prior to surgery and then randomly assigned into exercise and sedentary groups. The occluded-exercise (Occl-Ex; n=7) were endurance trained on a treadmill at 0% grade, beginning one week post-surgery; whilst the occluded-sedentary (Occl-Sed; n=5) were limited to cage activity within a 2 x 4 m pen. Occl-Ex pigs underwent progressive daily training sessions 5 days/week for 12 weeks. The training protocol included a warm-up prolonged running and cools down stages, where the durations and intensity of exercise bouts were increased as the animals were capable. Briefly, warm-up and cool down stages lasted for 5 min each at 1.5 mph. The endurance stage was running at a constant speed of 3.1 mph, building up to a total time of 75 min as the animals were capable. This intensity could easily be achieved by the occluded animals for approximately 75 min/session within approximately 9 weeks after beginning training. Exercise stress tests were conducted for both occluded groups, once every week by running on the treadmill beginning at 1mph and increasing the speed by 1mph each min until the pig was unable to continue. Blood pressure and heart rate were monitored during the stress tests. In addition, non-occluded pigs, age-matched, served as a control group (Non-Occl-Con; n=6); these pigs were not exercise neither trained nor given the stress test each week.

### In vitro assessment of vessel reactivity Vascular ring preparation

At the end of the 12 weeks of exercise training, pigs were anesthetized with intramuscular ketamine (35 mg/ kg)-xylazine (2.25 mg/kg), and intravenous thiopenthal (25 mg/ kg) for deep anesthesia, and the heart was removed to achieve euthanasia and placed in iced Krebs solution. The left anterior descending (LAD) and left circumflex (LCX) coronary arteries were removed from the heart. The arteries were trimmed of connective tissue and fat in cold Krebs bicarbonate buffer solution; and cut into six LAD and two LCX vessel rings each 3–4 mm in length. Cut rings were photographed on an Olympus SZH video microscope which was connected to a Spot Insight camera (model 3.2.0., Diagnostic Instruments, Inc., Sterling Heights, MI). Then, the rings morphological characteristics (axial length, outer and inner diameters) were measured using Image J software (1.34n, NIH, USA).

### Vascular rings set up

Six LAD and two LCX rings were used, for *in vitro* studies, and mounted on myographs (Globaltown Microtech., Inc., Sarasota, FL) by positioning two stainless steel wires in the lumen of each ring. Arterial rings were placed in a 20 mL bath of Krebs bicarbonate solution. The optimal point in the length-tension relationship (L_max_) was established through incremental (10% of passive outside diameter) increases in stretch in combination with potassium chloride (KCl: 50 mM) administration to the rings. The remainder of the study was performed at the experimentally determined L_max_ of each ring. Contractile responses to KCl were performed prior to vasorelaxation studies. KCl (80 mM) was administered twice to all of the arterial rings until the increase in tension had reached a plateau (10 min). Following the KCl experiments, Krebs bicarbonate solution was replaced every 20 min until resting tension was achieved.

### Vascular reactivity protocol I

It was designed to examine the relaxation responses. Endothelium-dependent, dose-dependent vasorelaxation was assessed in six LAD rings using cumulative addition of adenosine diphosphate (ADP; 10^−9^–10^−4^ M) and bradykinin (BK; 10^−11^–10^−6^ M); whilst the assessment of endothelium-independent vasorelaxation utilized increasing doses of sodium nitroprusside (SNP; 10^−10^–10^−4^ M). The LAD rings were preconstricted with prostaglandin F_2α_ (PGF_2α_; 30 μM) and allowed to achieve a plateau in tension development before the addition of vasodilators. For each pig’s heart ring one was left untreated (intact). Ring two was pretreated with nitro-L-arginine methyl ester (_L_-NAME; 300 mM) to inhibit the nitric oxide synthase (NOS) pathway. The third ring was pretreated with indomethacin (Indo; 5 mM) to inhibit cyclooxygenase (COX), the enzyme responsible for prostacyclin (PGI_2_) and prostaglandin production. Ring four was pretreated with a combination of _L_-NAME and Indo to assess the importance of NOS-and COX-independent mechanisms of relaxation. Ring five was pretreated with a combination of L-NAME, Indo, and potassium chloride (KCl) to block NOS, COX and the non-NOS, non-COX pathway, endothelium-derived hyperpolarizing factor (EDHF). Finally, the endothelium was removed from ring six; “denuded” by gentle rubbing of the luminal surface with fine-tipped forceps to study the endothelium- independent mechanisms, explicitly smooth muscle relaxation. Adequate ring denudation was tested by examining responses to maximal BK (30 mM). Ring six was considered to be properly denuded by the inability of BK to induce vasorelaxation (i.e. less than 5%) in PGF_2α_ (30 μM) preconstricted rings. The order of agonists throughout the entire study was ADP, BK, and SNP. Following each agonist induced dose response; Krebs bicarbonate solution was replaced at 20min intervals until resting tension of all arterial rings was reached (roughly 60min), before the next protocol was initiated.

### Vascular reactivity protocol II

The vasoconstrictor properties were assessed in two LCX rings using cumulative addition of phenylephrine (PE; 10^−2^–10^−7^ M), angiotensin II (Ang II; 10^−4^–10^−8^) and endothelin-1 (ET-1; 10^−5^–10^−7^ M). Ring one was left untreated; “intact”, whilst on ring two the endothelium was removed as described above; “denuded”.

### Solutions and drugs

The Krebs–bicarbonate buffer solution contained (in mM) 131.5NaCl, 5.0KCl, 1.2NaH_2_PO_4_, 1.2MgCl_2_, 2.5CaCl_2_, 11.2 glucose, 20.8NaHCO_3_, 0.003 propranolol, and 0.025 EDTA. Solutions were aerated with 95% O_2_ and 5% CO_2_ (pH 7.4) and maintained at 37°C. Angiotensin II was purchased from Bachem Americas (Torrance, CA), and all other drugs and chemicals were purchased from Sigma Chemical (St Louis, MO).

### Statistical analysis

All values are means ± SE. Differences among groups regarding ring characteristics, half-maximal effective concentration (EC_50_) values, and maximal relaxation responses were determined via one-way ANOVA with a Kruskal-Wallis test using Graph Pad Prism version 5.0a. Means of the EC_50_ values are presented as the negative log of the molar concentration. The analysis of concentration-response curves was performed using the mixed procedure in SAS version 9. The statistical model used was a three-factor analysis of variance with one between subjects factor (group; Non-Occl-Con, Occl-Ex, and Occl-Sed), and two within subject factors (treatment and dose). The Mixed procedure also allows us to model for heterogeneous variances across doses. Pairwise comparisons were made between groups for fixed treatment and dose levels using Least Squares Means. Nonparametric statistical methods were used to perform a series of tests on dose-response data in the cases where examination of residual plots from a three-factor analysis of variance model indicated that the assumption of normality of the error terms was suspect. Specifically, group differences at each dose-treatment combination were looked at with the Wilcoxon rank sum test. Treatment differences at each dose-group combination were also examined with the Wilcoxon signed-rank test on the differences. A false discovery rate adjustment was used for multiple tests in view of the large number of tests considered. Differences with false discovery rate-adjusted *P*-values of *P*<0.05 were considered significant.

## Results

### Animal characteristics

All Occl-Ex pigs completed the training program. The body weights were Non-Occl-Con, 27.5±0.74 kg; Occl-Ex, 39.4±2.2 kg; and Occl-Sed, 42.9±4.21kg. Although we included age-matched controls, the Non-Occl-Con were significantly smaller than both, Occl-Ex and Occl-Sed, (*P*<0.01). By the end of the training period, animals in the Occl-Ex group could run up to a higher intensity of 6–7 mph, as compared to only approximately 5mph for the Occl-Sed group during the weekly graded treadmill running test. In addition, Occl-Ex pigs showed a significantly lower peak blood pressure during the stress test than the Occl-Sed pigs (121±4 mmHg and 135±7 mmHg respectively (*P*<0.01). The peak heart rates were not significantly different between groups (Occl-Ex; 235±15 bpm, Occl-Sed; 255±10 bpm).

### Vessel characteristics

Structural and functional characteristics of rings harvested from LAD and LCX are presented in (Table 1). There were no significant differences in the physical or functional characteristics among the rings from all three groups, except for a smaller outer diameter and wall thickness (*P*<0.05) of the rings from the Non-Occl-Con pigs.

### Relaxation responses to BK

**Figure 1:**
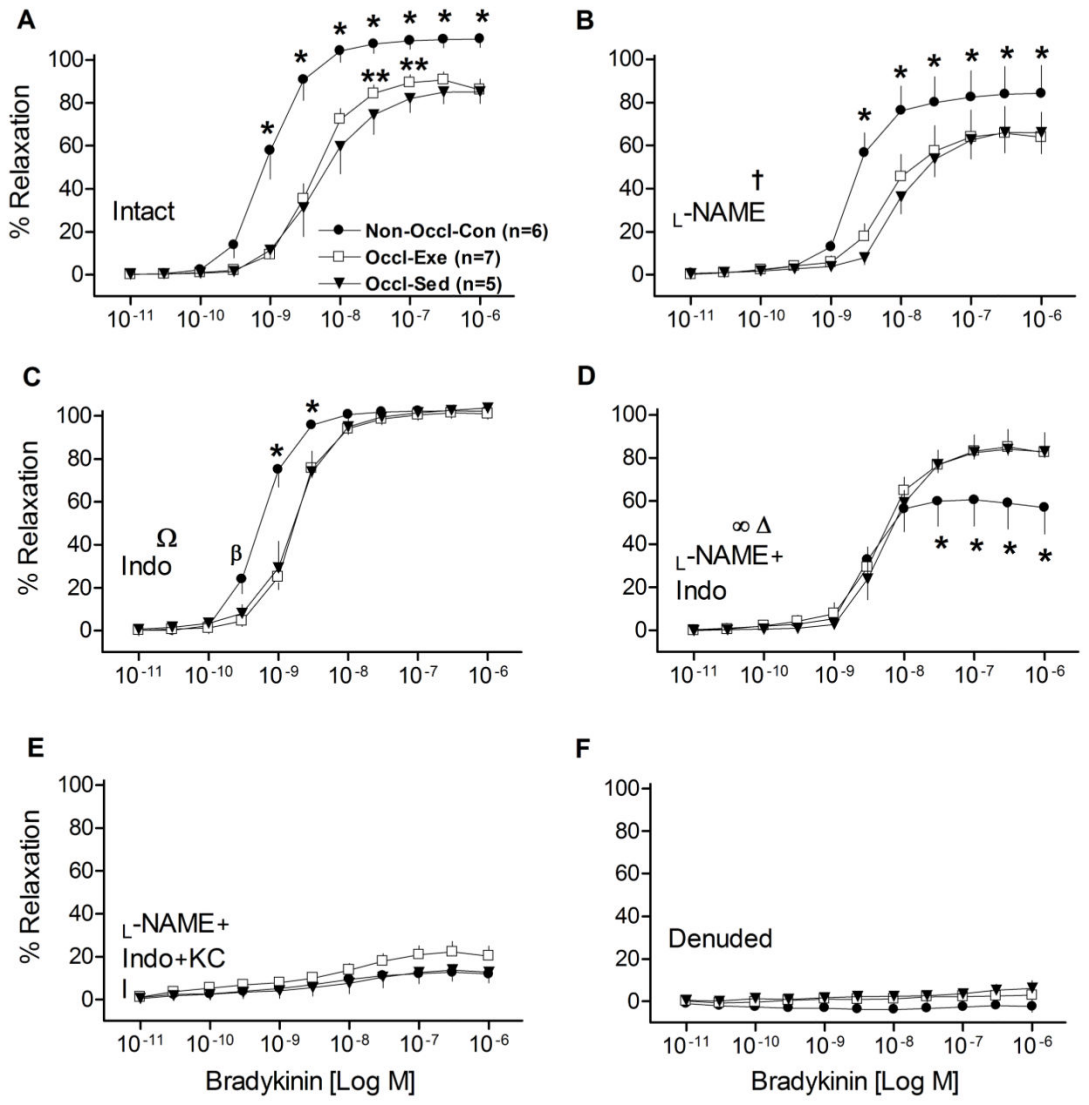
Concentration-response curves for bradykinin (BK)-induced relaxation of isolated left descending coronary artery rings from non-occluded control (Non-Occl-Con, n=6); occluded exercise trained (Occl-Ex, n=7), and occluded sedentary (Occl-Sed, n=5) pigs. Data were obtained for Intact vessels (panel A); in the presence of _L_-NAME (panel B); in the presence of Indo (panel C); in the presence of both, _L_-NAME + Indo (panel D); in the presence of _L_-NAME + Indo + 30mM KCl (panel E); and denuded vessels (panel F). Values are means ± SE. * Non-Occl-Con different from both Occl-Sed and Occl-Ex (P<0.001); β (P<0.05); ** Occl-Ex greater than Occl-Sed (P<0.001); ^†^_L_-Name decreased values in all groups; ^Ω^Indo increased values in Occl-Sed and Occl-Ex groups (P<0.001); ^∞^_L_-NAME + Indo reduced values of the Non-Occl-Con group (P<0.001), but not in the Occl-Sed or Occl-Ex groups, and eliminated the modest training improvement observed at the intermediate doses (cf., Figure 1 panel A); ^Δ^Addition of _L_-NAME + Indo reduced values in all groups (P<0.001).

BK-induced concentration-dependent relaxation in LAD rings was less (*P*<0.001) in intact rings from both Occl-Ex and Occl-Sed pigs than the Non-Occl-Con pigs (Fig. 1A). This reduced sensitivity to BK in the femoral occluded groups is also indicated by greater EC50 (*P*<0.05) by approximately a log-dose (Table 2). The Occl-Ex group exhibited a modest improvement in vasodilatation (*P*<0.001) in a dose-dependent manner, compared to the Occl-Sed group. BK-induced relaxation was partially inhibited by the addition of _L_-NAME in all three groups (*P*<0.001) (Fig. 1B); while the relative relationship among dose-response curves of the groups was not changed, the modest improvement at the two doses in the Occl-Ex group was eliminated suggesting the importance of NO in the Occl-Ex group. Addition of Indo to the baths increased relaxation in both Occl-Ex and Occl-Sed groups (*P*<0.001), suggesting the presence of a prostanoid constrictor in the occluded vessels (Fig. 1C). However, BK-induced relaxation of the Non-Occl-Con rings was more sensitive (*P*<0.001), indicating the presence of a COX-sensitive vasoconstrictor even in the absence of occlusion. With the combination of inhibitors, _L_-Name and Indo, BK-induced relaxation was reduced in all three groups (*P*<0.001), with a marked reduction in the Non-Occl-Con group such that the values were now below those of the Occl-Sed and Occl-Ex groups (*P*<0.001; Fig. 1D). BK-induced relaxation was almost completely inhibited by _L_-NAME + Indo + KCl in all three groups (Fig. 1E). Finally, when the endothelium was mechanically removed, BK-induced vasorelaxation was completely abolished in all three groups (Fig. 1F).

### Relaxation responses to ADP

**Figure 2:**
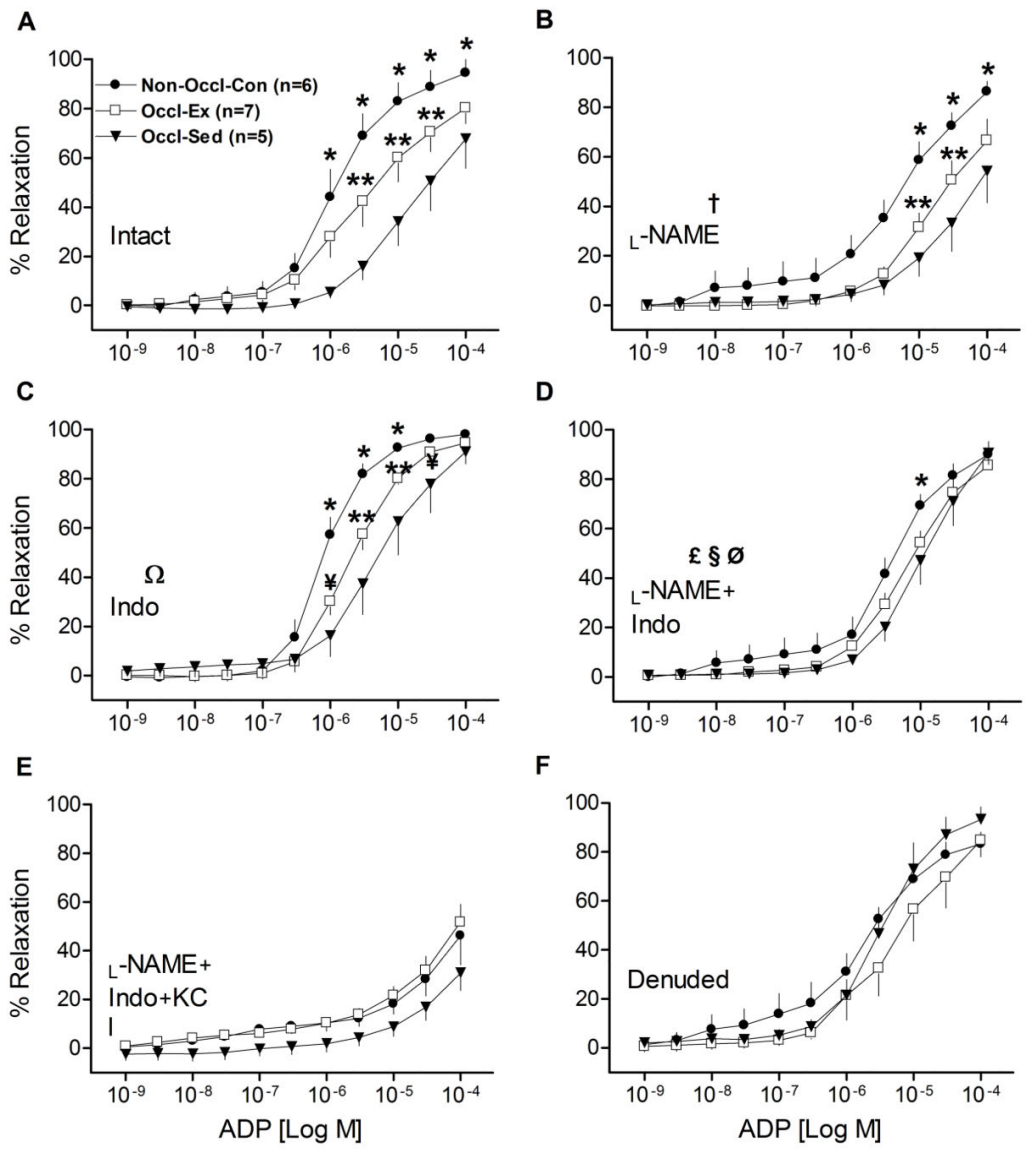
Concentration-response curves for adenosine diphosphate (ADP)-induced relaxation of isolated left descending coronary artery rings from non-occluded control (Non-Occl-Con, n=6); occluded exercise trained (Occl-Ex, n=7); and occluded sedentary (Occl-Sed, n=5) pigs. Data were obtained for Intact vesssels (panel A); in the presence of _L_-NAME (panel B); in the presence of Indo (panel C); in the presence of both, _L_-NAME + Indo (panel D); in the presence of _L_-NAME + Indo + 30 MM KCl (panel E); and denuded vessels (panel F). Values are means ± SE. *Non-Occl-Con greater than both Occl-Sed and Occl-Ex (p<0.001); ** Occl-Ex greater than Occl-Sed (P<0.001); ^¥^(P < 0.05); _L_-NAME decreased values in all groups (P<0.001); ^Ω^Indo increased values in all groups (P<0.001), in a dose-dependent manner, but retained the relative differences among groups; ^£^_L_-NAME + Indo eliminated the differences between groups (cf., panel A) by decreasing values of Non-Occl-Con group (P<0.001) and increasing values in Occl-Sed group (P<0.05) with dose-group interaction (p<0.001); ^§^_L_-NAME added to Indo decreased values in all groups (P<0.001); ^Ø^Indo added to _L_-NAME elevated values in Occl-Sed and Occl-Ex (P<0.001) and eliminated the training effect observed with _L_-NAME only.

As illustrated in (Fig. 2), ADP-induced relaxation was significantly reduced by femoral artery occlusion, as the relaxation curve for the intact Occl-Sed group coronary vessels was much less (*P*<0.001), and exhibited a greater EC_50_ (Table 3), than the Non-Occl-Con group. Interestingly, the ADP-induced relaxation curve of the Occl-Ex group (Fig. 2A) was intermediate (*P*<0.001) from the deficit of the Occl-Sed group. ADP-induced relaxation was in part inhibited by _L_-NAME in all groups (Fig. 2B), although the relative relationships among the curves remained. The response of the Occl-Sed group remained below (*P*<0.001) that of the Non-Occl-Con group with the relaxation curve of the Occl-Ex intermediate (*P*<0.05) in a dose-dependent manner (Fig. 2B). Further, there was an increase in dilatation in all groups (*P*<0.001), in a dose-dependent manner, when Indo was added to the bath (Fig. 2C). The Non-Occl-Con group was greater than both other groups (*P*<0.001) and the Occl-Ex group greater than the Occl-Sed group (*P*<0.05) in a dose-dependent manner. Consistently, the relative sensitivities to ADP, as evident in EC_50_ (Table 3), were unchanged by either _L_-NAME or Indo. However, in the presence of both _L_-NAME and Indo ADP-induced relaxation became similar among all three groups (Fig. 2D), due in part to decreasing values in the Non-Occl-Con group (*P*<0.001) and increasing values in the Occl-Sed group (*P*<0.05) with dose-group interactions (*P*<0.001). It should be recognized that the ADP-induced relaxation remained rather robust for all three groups (i.e., different from zero), indicating that pathways different from NOS and COX are important for vasorelaxation. In contrast, ADP-induced relaxation was markedly inhibited by _L_-NAME + Indo + KCl (Fig. 2E) leading to a reduced sensitivity to ADP (Table 3); however, some vasorelaxation remained implying that additional mechanisms might play a role. When the endothelium was removed from the LAD rings, ADP-induced relaxation was robust and similar among all three groups; this indicates that ADP works on both endothelial and vascular smooth muscle cells (Fig. 2F). Importantly, following removal of the endothelium there were no longer any differences in the responses of the three different groups indicating that all of the effects of femoral artery occlusion and exercise training were mediated through the endothelium.

### Relaxation responses to SNP

**Figure 3:**
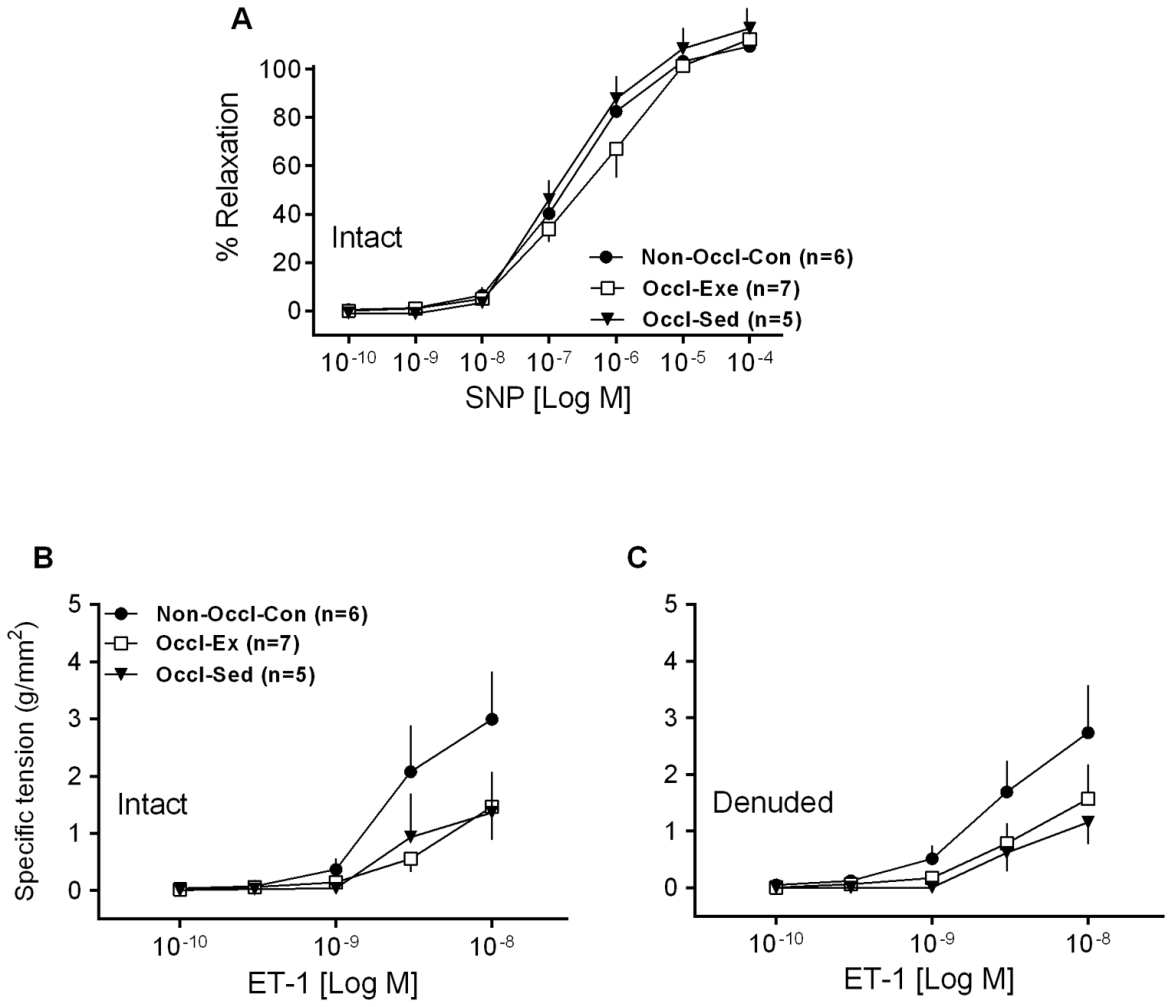
Concentration-response curves for Sodium nitroprusside (SNP)-induced relaxation of isolated left descending coronary artery rings from non-occluded control (Non-Occl-Con, n=6); occluded exercise trained (Occl-Ex, n=7) and occluded sedentary (Occl-Sed, n=7) pigs. Values are means ± SE. There were no significant differences among groups. B. and C. Concentration-response curves for Endothelin-1 (ET-1)-induced contraction of isolated left circumflex coronary artery rings from non-occluded control (Non-Occl-Con, n=6); occluded exercise trained (Occl-Ex, n=7) and occluded sedentary (Occl-Sed, n=7) pigs. Values are means ± SE per square millimeter of artery wall. There were no significant differences among groups.

Sensitivity to an NO donor was not different among groups, as SNP responses used to assess endothelium-independent relaxation in LAD rings were not significantly different among Non-Occl-Con, Occl-Ex and Occl-Sed pigs (Fig. 3A).

### Contractile responses

In order to evaluate the vasoconstrictor response of coronary vessels, isometric tension studies were performed in isolated LCX rings using ET-1. The dose responses to ET-1 for intact and denuded vessels (Fig. 3, B&C) were not significantly different among groups. Exercise training did not modify the vasoconstriction to ET-1. Similar results were observed with, PE, and Ang II (data not shown).

## Discussion

The present study was designed to test the hypothesis that swine with bilateral occlusion of the femoral arteries would exhibit coronary endothelial dysfunction. In addition, we anticipated that exercise training would be able to preserve and/or improve coronary endothelial function. We report here that simple femoral artery occlusion resulted in significantly decreased BK-and ADP-induced coronary endothelium-dependent relaxation, as compared to Non-Occl-Con pigs. This is to our knowledge the first observation that occlusion of the distant hindlimb arteries would lead to dysfunction of vessels within a central vascular bed such as coronary conduit vessels. Furthermore, exercise training partially ameliorated the coronary dysfunction, particularly with ADP. Thus, it is likely that daily physical activity can help ameliorate the coronary dysfunction induced by PAI.

### Influence of PAI on coronary vasoresponsiveness

The finding that femoral artery occlusion markedly decreased coronary relaxation (Figs. 1A & 2A) was consistent with our hypothesis. The reason for this coronary dysfunction appears to be somewhat complex. First, BK- and ADP-induced relaxations were significantly inhibited in all three groups (Figs. 1B & 2B) by the addition of the NOS inhibitor _L_-NAME. However, the deficit in vasorelaxation in the occluded groups (Occl-Sed and Occl-Ex) remained. This implies that some non-NOS pathway contributes to the coronary dysfunction caused by occlusion of the femoral arteries. Further, the lack of differences in the SNP-induced responses among groups indicates that the responsiveness of the smooth muscle to NO is not an important factor underpinning the reductions in vessel relaxation observed with intact vessels from the occluded groups. Second, significant improvements in BK- & ADP-induced relaxation became evident with inhibition of the COX pathways with Indo (Figs. 1C & 2C), implicating the importance of vasoconstrictor substances, likely prostanoids, especially in the occluded groups. However, significant deficits in BK- and ADP-induced relaxations were still present in the rings of the Occl-Sed and Occl-Ex animals in the presence of Indo. This indicates that some non-COX pathway contributes to the coronary dysfunction cause by occlusion of the femoral arteries. Third, when both Indo and _L_-NAME were administered the deficits in vasodilation in the occluded groups, as compared to the Non-Occl-Con group, were essentially eliminated (Figs. 1D & 2D). This implies that a potential interaction among NOS and COX pathways, which are known to exist^[26]^, is responsible for the deficit in BK-induced coronary dilation caused by occlusion of the femoral arteries. However, it is important to note that the dilatory responses were substantial in all groups with NOS and COX inhibition, suggesting non-NOS non-COX pathways (likely endothelial-derived hyperpolarizing factor; EDHF) contribute to vasorelaxation in coronary arteries. Indeed, it has been reported that EDHF-mediated responses play an important role in conduit arteries for endothelium-dependent relaxation, especially the coronary vascular bed^[27]^. The observation that BK-stimulated dilatory responses of the occluded groups were actually greater (Fig. 1D) than that of the Non-Occl- Con group implies that the putative EDHF was more dominant in the occluded animals. Consistent with this interpretation, the addition of KCl, which acts as EDHF inhibitor, in combination to L-NAME and Indo markedly blunted BK- and ADP-induced relaxation (Figs. 1E & 2E) and eliminated the effect of occlusion observed with BK (Fig. 1D). The fact that ADP-induced significant relaxation in denuded LADs (Fig. 2F) indicates that receptors located in vascular smooth muscle of LAD rings also must be responsive. ADP can induce vasodilation via nucleoside-selective P_2Y1_ receptors (“purinoceptors”) located on vascular smooth muscle cells of porcine coronary arteries^[28]^. However, the fact that the dilatory responses were similar in Non-Occl- Con, Occl-Sed, and Occl-Ex animals after denudation (Fig. 2F) indicates that the reduced responses to ADP, caused by femoral artery occlusion (Fig. 2A) are the result of endothelium-dependent processes. Thus, we interpret these findings to indicate that occlusion of the femoral arteries causes significant coronary dysfunction that is endothelial in origin and not dependent solely on the NOS nor COX pathways, but implicates another pathway purported to an EDHF.

### Influence of exercise training on PAI-induced vascular dysfunction

Similar to the exercise training effects apparent in the coronary vessels of normal, non-occluded pigs observed previously^[ 29,30]^, the present study demonstrates that exercise training can significant ameliorate coronary vessel dysfunction caused by femoral artery occlusion (Figs. 1A & 2A). The use of two dilating agents (BK and ADP) provided both confirming and somewhat different responses. For example, the significantly improved vasodilatation in intact coronary rings, of the Occl-Ex group as compared to the Occl-Sed group (*P*<0.001), that was evident with both BK and ADP, was eliminated by the inhibition of both the NOS and COX pathways with _L_-NAME and Indo, respectively. This implies that either or both NOS and COX pathways are altered by exercise training and contribute to the improved vasoresponsiveness by an enhanced NO-dependent vasorelaxation and/or a reduced COX-dependent vasoconstrictor effect. On one hand, the differences between the Occl-Ex and Occl-Sed groups (Figs. 1A & 2A) were eliminated by either NOS inhibition or COX inhibition with BK-induced vasodilatation (Fig. 1, B & C), but remained significantly different with ADP-induced vasodilatation (Fig. 2, B & C). This greater ADP-induced dilatation with exercise training apparent with either _L_-NAME or Indo added to the vessels implies that neither the NOS nor COX pathway is essential for the exercise training effect. However, this view does not recognize the potential interaction that can occur among vasoactive pathways in coronary vessels^[31,32,26,33]^. For example, the interactions between vasodilatory and vasoconstrictory responses become evident with interactions between the NOS and COX pathways^[34]^. Indeed, Bagi, Ungvari and Koller^[34]^ observed that in the presence of excess of superoxide and NO peroxynitrite production is enhanced leading to an enhanced COX-dependent production of vasoconstriction. Such an interaction among the NOS and COX pathways could have contributed to the improved dilatory response observed with ADP in the Occl-Ex, as compared to Occl-Sed animals (Fig. 2, B & C), in the presence of _L_-NAME (leading to an a reduced COX-dependent vasoconstriction) or in the presence of Indo (leading to an enhanced NOS-dependent vasodilatation). It is unclear why this was not observed with BK (Fig. 1 B & C); whether a differential influence of ADP and BK on COX activity could have contributed to the failure to observe the training effect with BK in the presence of either _L_-NAME or Indo is speculation. We interpret our findings to indicate that exercise training can help ameliorate the vasodilatory deficit induced by occlusion of the femoral arteries. The basis for this response includes an enhanced NO bioavailability and possibly an interaction among NOS and COX pathways.

### Influence of PAI on contractile responses

There were no significant differences among the responses to ET-1 (Fig. 3, B & C), PE, or Ang II (data not shown) of LCXs from Occl-Ex and Occl-Sed *vs* Non-Occl-Con. In addition, the 80 mM KCl contractile responses (i.e., absolute and relative tension) (Table 1) were not significantly different in LCXs among the groups. Similarly, the vasoconstrictor effects of 80 mM KCl and PGF_2α_ (Table 1) were also not different in LADs from the three groups of pigs. Previous studies have emphasized that ET-1 may play an important role in the alteration of endothelial function at the onset of PAD^[35]^. Truly, PAD patients with concomitant CAD exhibit excessive plasma production of ET-1, especially in the initial stages of the disease^[35]^. Furthermore, the vascular smooth muscle in patients with peripheral atherosclerotic disease also develops functional abnormalities, causing leg conduit arteries to be unrespon¬sive to vasodilators and vasoconstrictors, leading to impaired blood flow to the lower limb and contributing to symptoms of claudication^[29]^. These considerations lead us to propose that bilateral femoral artery occlusion, in our PAI model, would also promote vasoconstrictor abnormalities reflected in altered contractile responses of LCXs. The findings of the present investigation did not support this hypothesis. However, it is important to emphasize that perhaps the lack of altered ET-1 responses might be due to the fact that our young Yucatan pigs were not encumbered by atherosclerosis, in contrast to PAD patients with concomitant CAD.

### PAI and endothelial coronary dysfunction

Preclinical animal models of PAI, similar to that used in this experiment, have been well characterized and extensively used to investigate potential therapeutic approaches to treat patients with PAD^[18]^. A major advantage of this model is that it exhibits limb blood flow at rest that is minimally altered, but during exercise blood flow becomes inadequate, characteristic of that seen in patients with intermittent claudication^[18,19]^. On the other hand, the typical limitations of these models, in not simulating human PAD (e.g., acute onset of vessel occlusion, use of healthy young animals, absence of atherosclerotic diseases vessels, etc.) may actually be an advantage in the present experiment as it potentially eliminates confounding factors that often present as comorbidities in human PAD patients. Thus, while patients with intermittent claudication can exhibit endothelial dysfunction^[5,6]^ and an increased inflammatory status^[36]^ with impairment of the coronary arteries^[9]^ it is difficult to assess the effects of PAI as an independent cause of any central vascular dysfunction. In contrast, in the present study we are able to evaluate distinct outcomes of ‘simple’ PAI in relatively young pigs that are free from atherosclerotic/inflammatory disease. Certainly, Yucatan miniature swine are highly resistant to frank atherogenesis. Minimal signs of atherogenesis are only evident after feeding them an exceptionally high-fat diet, representing 46% of their daily caloric intake, for an extended period of time (20–24 weeks)^[22–24]^. Even then the extent of atherosclerosis in central and peripheral arteries is relatively modest characterized by a Stary Stage I-III classification^[22,24,25]^, similar to that reported in studies of early human atherosclerosis^[37]^. Thus, our experiment could expose consequences of PAI that are manifest, even in the absence of extensive comorbidities.

The most important finding of the present study is that coronary artery endothelial dysfunction was observed in other- wise healthy pigs following bilateral occlusion of the femoral arteries. Although the precipitating cause of this dysfunction is presently unclear, a couple of contributing factors can be proposed. First, it is now well recognized that active muscle, even in normal healthy humans, releases an abundance of potent cytokines, including factors potentially involved in the inflammatory process^[38]^. This appears to be exaggerated in PAD patients, as ischemia induced by exercise promotes oxidative stress and inflammation^[30]^ that has been linked to an attenuation of endothelium-dependent vascular smooth muscle relaxation^[26]^. Accordingly, we could speculate that the decreased coronary vasorelaxation reported in this study might be the consequence of altered NO availability induced by excessive oxidative stress even with simple cage activity in all of the occluded pigs. However, our results indicate occlusion promulgates changes in signal pathways far more extensive than the NOS. On the other hand, it is apparent that chronic physical activity, similar to that experienced by our trained pigs, exerts an anti-inflammatory effect^[4,38]^. Elevated biomarkers of chronic inflammation are reduced by exercise training in patients with chronic cardiovascular disease^[4]^, including PAD^[39,40]^. If this occurred in our Occl-Ex pigs, it could have contributed to the ameliorated dilatory dysfunction observed in the coronary vessels with exercise training. Second, there is the potential that PAI leads to a hypersympathetic state. Sympathetic nervous activity (SNA) is expected to increase in response to exercise (i.e., muscle contractions) and is exacerbated during ischemic exercise^[41]^. In PAD patients there is an exaggerated increase in blood pressure during walking^[34]^that is related to walking speed and duration, as well as the severity of disease^[42,34]^. This increased blood pressure has been attributed to an enhanced sympathetic output through the ischemic pressor response^[41,42,4]^. Similar increases in mean arterial blood pressure, attributed to an observed increase in SNA, have been reported in studies using animal models of PAI^[43]^. An increase in SNA during activity could have contributed to our results, as the associated increase in blood pressure driven by an enhanced SNA is known to be a cardiovascular risk factor leading to a reduced endothelial dependent vasodilatation^[44]^. Interestingly, it has been reported that exercise training can normalize SNA, alleviate levels of vasoactive substances leading to vasoconstriction^[45]^, and promote a reduction in systemic blood pressure^[16]^. Consistent with these observations, our data showed that Occl-Ex pigs exhibited significantly lower peak blood pressure during the stress test than the Occl-Sed pigs (121±4 mmHg and 135±7 mmHg respectively; *P*<0.01).

In conclusion, we report here that bilateral occlusion of the femoral arteries, used to establish a preclinical model of PAI, induces coronary endothelial dysfunction, evident as a reduced vasodilatory responsiveness. These observations are potentially most significant in view of the sizeable number of PAD patients that are free from frank CAD^[12–14]^ representing approximately 35 to 50% of all patients with PAD^[10,11]^. Further, exercise training partially ameliorated the dilatory deficit. The potential that muscle- derived cyotkines/factors and/or a hypersympathetic state and their amelioration by training contributed to these responses remains to be determined. To the extent that our findings apply to these human patients, this implies that individuals with PAD are at risk of coronary dysfunction, even in the absence of generalized atherosclerotic disease that affects central vessels.

## Figures and Tables

**Table 1:** Vessels characteristics.

Variable	Non-Occl-Con (n=6)	Occl-Sed (n=5)	Occl-Ex (n=7)
**LAD rings**			
Axial length, mm	3.78 ± 0.12	3.38 ± 0.14	3.66 ± 0.25
Outer diameter, mm	2.14 ± 0.15*†	2.84 ± 0.20	2.87 ± 0.18
Inner diameter, mm	1.24 ± 0.10	1.58 ± 0.14	1.55 ± 0.16
Wall thickness, mm	0.46 ± 0.03*†	0.63 ± 0.05	0.66 ± 0.03
Resting tension, g	5.82 ± 0.64	7.85 ± 1.15	9.66 ± 2.11
80 mM KCl tension, g	20.45 ± 2.35	24.37 ± 3.87	22.60 ± 2.04
80 mM KCl specific tension, g/mm^2^	4.20 ± 0.38	3.87 ± 0.76	3.11 ± 0.45
PGF_2α_ tension, g	20.38 ± 2.52	24.85 ± 3.71	23.44 ± 2.19
**LCX rings**			
Axial length, mm	3.75 ± 0.23	3.95 ± 0.04	3.81 ± 0.19
Outer diameter, mm	1.84 ± 0.19†	2.04 ± 0.11	2.70 ± 0.15
Inner diameter, mm	1.01 ± 0.09	0.88 ± 0.11	1.18 ± 0.13
Wall thickness, mm	0.42 ± 0.06†	0.58 ± 0.01	0.76 ± 0.11
Resting tension, g	6.42 ± 0.94	6.93 ± 1.01	5.53 ± 0.89
80 mM KCl tension, g	17.44 ± 2.40	23.04 ± 2.65	19.77 ± 4.34
80 mM KCl specific tension, g/mm^2^	3.55 ± 0.60	4.43 ± 0.35	2.89 ± 1.09

Values are means ± SE. Vessels were obtained from non-occluded control (Non-Occl-Con), occluded sedentary (Occl-Sed), and occluded exercise trained (Occl-Ex) pigs. Left anterior descending artery (LAD); left circumflex artery (LCX); potassium chloride (KCl); prostaglandin F_2α_ (PGF_2α_).

* P<0.05 for Non-Occl-Con vs. Occl-Sed;

† P<0.05 for Non-Occl-Con vs. Occl-Ex comparison at the measured characteristic.

**Table 2:** Maximal relaxation and EC_50_ values for BK-induced relaxation of LAD rings

Variable	Non-Occl-Con (n=6)	Occl-Sed (n=5)	Occl-Ex (n=7)
Intact			
Maximum relaxation, %	109.7 ± 3.85*†	85.80 ± 5.46	90.91 ± 3.71
EC_50_, - log M	−9.07 ± 0.15*†	−8.05 ± 0.26	−8.26 ± 0.11
_L_-NAME			
Maximum relaxation, %	84.55 ± 12.75	67.56 ± 9.31	66.19 ± 12.31
EC_50_, - log M	−8.09 ± 0.50	−7.23 ± 0.35	−7.27 ± 0.43
Indo			
Maximum relaxation, %	102.60 ± 1.57	103.70 ± 2.54	101.60 ± 0.65
EC_50_, - log M	−9.25 ± 0.08†	−8.80 ± 0.15	−8.76 ± 0.06
_L_-NAME + Indo			
Maximum relaxation, %	63.34 ± 12.16†	84.49 ± 2.88	85.85 ± 7.90
EC_50_, - log M	−7.41 ± 0.54	−8.03 ± 0.12	−7.95 ± 0.32
_L_-NAME + Indo + KCl			
Maximum relaxation, %	12.92 ± 2.87	13.93 ± 5.28	22.59 ± 4.84
EC_50_, - log M	−5.21 ± 0.18	−5.23 ± 0.22	−5.57 ± 0.20

Values are means ± SE. They were obtained from non-occluded-control (Non- Occl-Con), occluded sedentary (Occl-Sed), and occluded exercise trained (Occl- Ex) pigs. Half-maximal effective concentration (EC_50_); potassium chloride (KCL).

* P < 0.05 for Non-Occl-Con vs. Occl-Sed;

† P < 0.05 for Non-Occl-Con vs. Occl-Ex.

**Table 3:** Maximal relaxation and EC50 values for ADP-induced relaxation of LAD rings.

Variable	Non-Occl-Con (n=6)	Occl-Sed (n=5)	Occl-Ex (n=7)
Intact			
Maximum relaxation, %	94.41 ± 5.53	67.67 ± 11.92	76.42 ± 6.15
EC_50_, - log M	−5.78 ± 0.17*	−4.52 ± 0.29	−5.24 ± 0.26
_L_-NAME			
Maximum relaxation, %	86.27 ± 4.16	54.23 ± 12.77	66.54 ± 8.72
EC_50_, - log M	−5.32 ± 0.28*	−4.11 ± 0.31	−4.49 ± 0.15
Indo			
Maximum relaxation, %	98.02 ± 1.05	91.01 ± 4.98	94.62 ± 1.30
EC_50_, - log M	−6.03 ± 0.11*	−5.25 ± 0.29	−5.61 ± 0.08
_L_-NAME + Indo			
Maximum relaxation, %	89.93 ± 5.24	90.50 ± 4.59	85.69 ± 6.85
EC_50_, - log M	−5.41 ± 0.21	−4.93 ± 0.16	−5.03 ± 0.12
_L_-NAME + Indo + KCl			
Maximum relaxation, %	46.17 ± 11.83	30.91 ± 7.24	51.75 ± 7.29
EC_50_, - log M	−4.00 ± 0.21	−3.65 ± 0.20	−4.15 ± 0.14
Denuded			
Maximum relaxation, %	83.30 ± 4.82	93.23 ± 5.08	84.66 ± 6.67
EC_50_, - log M	−5.59 ± 0.23	−5.41 ± 0.21	−5.09 ± 0.30

Values are means ± SE. They were obtained from non-occluded control (Non- Occl-Con), occluded sedentary (Occl-Sed), and occluded exercise trained (Occl- Ex) pigs. Half-maximal effective concentration (EC_50_); potassium chloride (KCl).

* P<0.05 for Non-Occl-Con vs Occl-Sed.
